# GC-MS Studies on Derivatization of Creatinine and Creatine by BSTFA and Their Measurement in Human Urine

**DOI:** 10.3390/molecules26113206

**Published:** 2021-05-27

**Authors:** Olga Begou, Kathrin Weber, Bibiana Beckmann, Dimitrios Tsikas

**Affiliations:** Institute of Toxicology, Core Unit Proteomics, Hannover Medical School, 30625 Hannover, Germany; mpegolga@chem.auth.gr (O.B.); kathrin.weber89@gmx.net (K.W.); beckmann.bibiana@mh-hannover.de (B.B.)

**Keywords:** BSTFA, creatine, creatinine, derivatization, quantification, silylation, TMS, validation

## Abstract

In consideration of its relatively constant urinary excretion rate, creatinine (2-amino-1-methyl-5*H*-imidazol-4-one, MW 113.1) in urine is a useful endogenous biochemical parameter to correct the urinary excretion rate of numerous endogenous and exogenous substances. Reliable measurement of creatinine by gas chromatography (GC)-based methods requires derivatization of its amine and keto groups. Creatinine exists in equilibrium with its open form creatine (methylguanidoacetic acid, MW 131.1), which has a guanidine and a carboxylic group. Trimethylsilylation and trifluoroacetylation of creatinine and creatine are the oldest reported derivatization methods for their GC-mass spectrometry (MS) analysis in human serum using flame- or electron-ionization. We performed GC-MS studies on the derivatization of creatinine (d_0_-creatinine), [*methylo*-^2^H_3_]creatinine (d_3_-creatinine, internal standard) and creatine (d_0_-creatine) with *N*,*O*-*bis*(trimethylsilyl)trifluoroacetamide (BSTFA) using standard derivatization conditions (60 min, 60 °C), yet in the absence of any base. Reaction products were characterized both in the negative-ion chemical ionization (NICI) and in the positive-ion chemical ionization (PICI) mode. Creatinine and creatine reacted with BSTFA to form several derivatives. Their early eluting *N*,*N*,*O*-*tris*(trimethylsilyl) derivatives (8.9 min) were found to be useful for the precise and accurate measurement of the sum of creatinine and creatine in human urine (10 µL, up to 20 mM) by selected-ion monitoring (SIM) of *m*/*z* 271 (d_0_-creatinine/d_0_-creatine) and *m*/*z* 274 (d_3_-creatinine) in the NICI mode. In the PICI mode, SIM of *m*/*z* 256, *m*/*z* 259, *m*/*z* 272 and *m*/*z* 275 was performed. BSTFA derivatization of d_0_-creatine from a freshly prepared solution in distilled water resulted in formation of two lMate-eluting derivatives (14.08 min, 14.72 min), presumably creatinyl-creatinine, with the creatininyl residue existing in its enol form (14.08 min) and keto form (14.72 min). Our results suggest that BSTFA derivatization does not allow specific analysis of creatine and creatinine by GC-MS. Preliminary analyses suggest that pentafluoropropionic anhydride (PFPA) is also not useful for the measurement of creatinine in the presence of creatine. Both BSTFA and PFPA facilitate the conversion of creatine to creatinine. Specific measurement of creatinine in urine is possible by using pentafluorobenzyl bromide in aqueous acetone.

## 1. Introduction

**Scheme 1:**
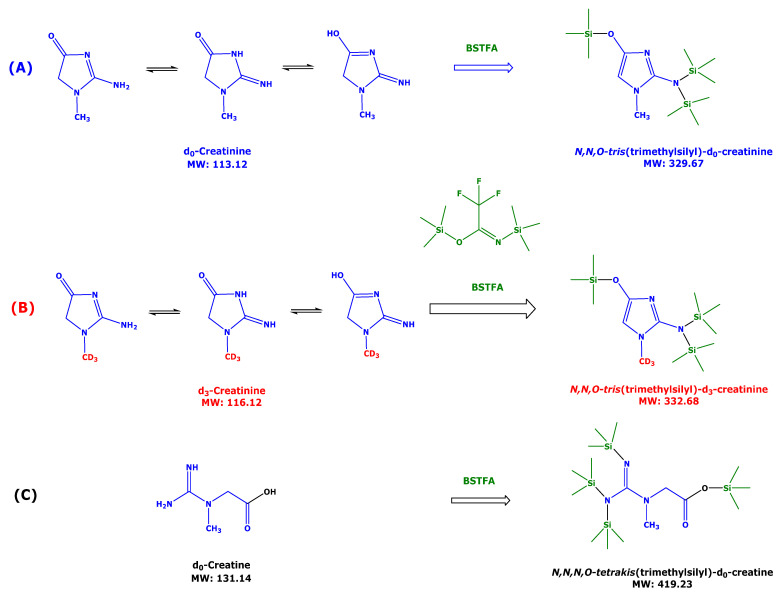
Schematic of the expected derivatization reaction and products of (**A**) unlabeled creatinine (d_0_-creatinine), (**B**) deuterium-labelled creatinine ([*methylo*-^2^H_3_]creatinine, d_3_-creatinine) and (**C**) unlabeled (d_0_-creatine) and with *N*,*O*-*tris*(trimethylsilyl)trifluoroacetamide (BSTFA) to form their *N*,*N*,*O*-*tris*(trimethylsilyl) creatinine derivatives (**A**,**B**) and *N*,*N*,*N*’,*O*-tetrakis(trimethylsilyl creatine derivative (**C**).

Creatinine (2-amino-1,5-dihydro-1-methyl-4*H*-imidazol-4-one, MW 113.12; see Scheme 1) is the end-product of creatine catabolism. Creatinine is excreted in the urine with a fairly constant rate and is generally used for the correction of renal excretion rates of endogenous and exogenous substances. This correction is indispensable in clinical studies when urine specimens from spontaneous micturition must be analyzed [1]. The mean concentration of creatinine in urine samples of healthy adults is approximately 12–13 mM, with men excreting higher amounts of creatinine than women [1]. Besides the spectrophotometric method based on the famous Jaffé reaction [2] many different analytical methods are currently available for creatinine. They include spectrophotometric, enzymatic and instrumental methods based on HPLC, GC-MS, LC-MS and LC-MS/MS [3,4,5,6,7,8,9,10,11,12,13,14,15,16,17,18,19]. Lawson [20] and Siekmann [21] demonstrated by electron ionization (EI) that creatinine reacts with silylation reagents to form its *N*,*N*,*O*-*tris*(trimethylsilyl) derivative. Björkhem and colleagues used trifluoroacetic anhydride for the derivatization of creatinine and its GC-MS analysis [22]. Trimethylsilylation derivatization reactions used in MS-based methods were found to be associated with interferences due to formation of several derivatives [23]. To our knowledge, the GC-MS measurement of urinary creatinine as *N*,*N*,*O*-*tris*(trimethylsilyl) derivative by negative-ion chemical ionization (NICI) or positive-ion chemical ionization (PICI) has not been reported thus far.

In the present study, we investigated in detail the derivatization of unlabelled creatinine (d_0_-creatinine), commercially available [methylo-^2^H_3_]creatinine (d_3_-creatinine) and unlabelled creatine (d_0_-creatine) by *N*,*O*-*bis*(trimethylsilyl)trifluoroacetamide (BSTFA), one of the oldest trimethylsilylation reagents for amino acids [24]. It is well known that BSTFA reacts with many functional groups, notably hydroxyl, carboxyl and amine groups. Based on this knowledge we expected that BSTFA will react with creatinine and creatine to form *N*- and *O*-derivatives (Scheme 1).

In our study, we used GC-MS in the NICI and in the PICI mode, confirmed the formation of the expected *N*,*N*,*O*-*tris*(trimethylsilyl) derivatives and identified several derivatives of creatinine and creatine that have not reported thus far. As creatinine and creatine are in a pH-dependent equilibrium and inter-convertible, our results suggest that BSTFA and GC-MS are not specific for creatinine and creatine but allow measurement of their sum. Using d_3_-creatinine as the internal standard we demonstrate that creatinine can be reliably quantitated in 10-µL aliquots of human urine by GC-MS as *N*,*N*,*O*-*tris*(trimethylsilyl) derivative with minimum labour.

## 2. Materials and Methods

### 2.1. Chemicals and Materials

Unlabeled creatine (d_0_-creatine), unlabeled creatine phosphate 4×H_2_O, unlabeled creatinine (d_0_-creatinine) and trideuterocreatinine, i.e., [methylo-^2^H_3_]creatinine (d_3_-creatinine; declared isotopic purity of >99 atom% ^2^H) were obtained from Aldrich (Steinheim, Germany). Stock solutions of d_0_-creatine, d_0_-creatinine and d_3_-creatinine (each 20 mM) were prepared in deionized water and stored in a refrigerator at 8 °C. BSTFA was obtained from Macherey-Nagel (Düren, Germany). Glassware for GC–MS (i.e., 1.5 mL autosampler glass vials and 0.2 mL microvials) and a fused-silica capillary column Optima 17 (15 m × 0.25 mm I.D., 0.25 µm film thickness) were purchased from Macherey-Nagel.

### 2.2. Derivatization Procedure for Creatinine in Human Urine Samples

Urine samples used in method development and validation were obtained from healthy volunteers being members of the researcher group and authors of this manuscript. Urine samples (1-mL aliquots) were kept frozen at −18 °C until analysis. Prior to sample derivatization, urine samples were thawed and centrifuged (5800× *g*, 5 min). Urine (10 µL) and synthetic creatinine-containing samples (usually 10 µL) were evaporated to complete dryness under a stream of nitrogen. Subsequently, the samples were treated with 100 µL absolute ethanol and the solvents were evaporated to dryness by a stream nitrogen gas to remove remaining water. Then, the residues were reconstituted with pure BSTFA (100 µL), the glass vials were tightly closed and heated for 60 min at 60 °C in a thermostat. After cooling to room temperature, aliquots (about 90 µL) were transferred into 1.8-mL autosampler glass vials equipped with 200-µL microinserts. Aqueous solutions (usually 10 µL aliquots) of creatinine and creatine were derivatized as described above.

### 2.3. GC–MS Conditions

In this work, we used a GC-MS method previously used in our group for amino acid derivatives [25]. GC-MS analyses were performed on a single-quadrupole mass spectrometer model ISQ directly interfaced with a Trace 1310 series gas chromatograph equipped with an autosampler AS 1310 from ThermoFisher (Dreieich, Germany). The following oven temperature program was used with helium as the carrier gas at a constant flow rate of 1 mL/min: 0.5 min at 40 °C, then increased to 210 °C at a rate of 15 °C/min and to 320 °C at a rate of 35 °C/min, respectively, and held at 320 °C for 1 min. Interface, injector and ion-source were kept at 300 °C, 280 °C and 250 °C, respectively. Electron energy was set to 70 eV and electron current to 50 µA. Methane (2.4 mL/min) was used as the reagent gas for NICI and PICI. Aliquots (1 µL from derivatization mixtures) were injected in the splitless mode by means of the autosampler using a 10-µL Hamilton needle, which was cleaned automatically three times with toluene (5 µL) after each injection. Quantitative analyses were performed in the selected-ion monitoring (SIM) mode. The peak area (PA) values of d_0_-creatinine and d_3_-creatinine were calculated automatically by the GC–MS software (Xcalibur and Quan Browser). The concentration of d_0_-creatinine was calculated by multiplying the peak area ratio (PAR) of d_0_-creatinine to d_3_-creatinine with the concentration of d_3_-creatinine added to the sample. Statistical analyses and graphs were performed and prepared by GraphPad Prism 7 (San Diego, CA, USA).

### 2.4. HPLC Analysis of Creatine, Creatinine and Creatine-Phosphate in HCl Solutions

We used a HPLC method previously reported by our group for creatinine measurement in human urine [16]. HPLC analyses were carried out on the an HPLC system consisting of an Agilent 1100 Series binary pump G1312A, an Agilent 1100 Series Degaser G1322A, an Agilent 1100 Series oven column Colcom G1316A, an Agilent 1100 Series VWD detector (all Agilent, Waldbronn, Germany, and an MP3 autosampler (Gerstel, Mülheim, Germany), ChemStation for LC-Systems Rev.B.0402SP1 (212) and Gerstel Maestro Version 1.3.20.41.13.5 were used to control the HPLC system and evaluate the analyses. HPLC analyses were performed on a Kinetex 5 µm EVO C18 100 Å column (250 × 4.6 mm) from Phenomenex (Aschaffenburg, Germany) at a fixed column temperature of 20 °C. The mobile phase was 100 mM sodium acetate, pH 7.5, 10 vol% methanol and was pumped at a flow rate of 1.0 mL/min. Samples (20 µL) were injected by means of the autosampler. The effluent was monitored at 210 nm. The analysis time was 5 min. The retention time was 2.073 ± 0.018 min for creatine-phosphate, 2.252 ± 0.007 min for creatine and 2.547 ± 0.007 min for creatinine.

## 3. Results

### 3.1. Generation of GC-MS Spectra and Characterization of Derivatization Products of d_0_-Creatine and of d_3_-Creatinine

**Figure 1:**
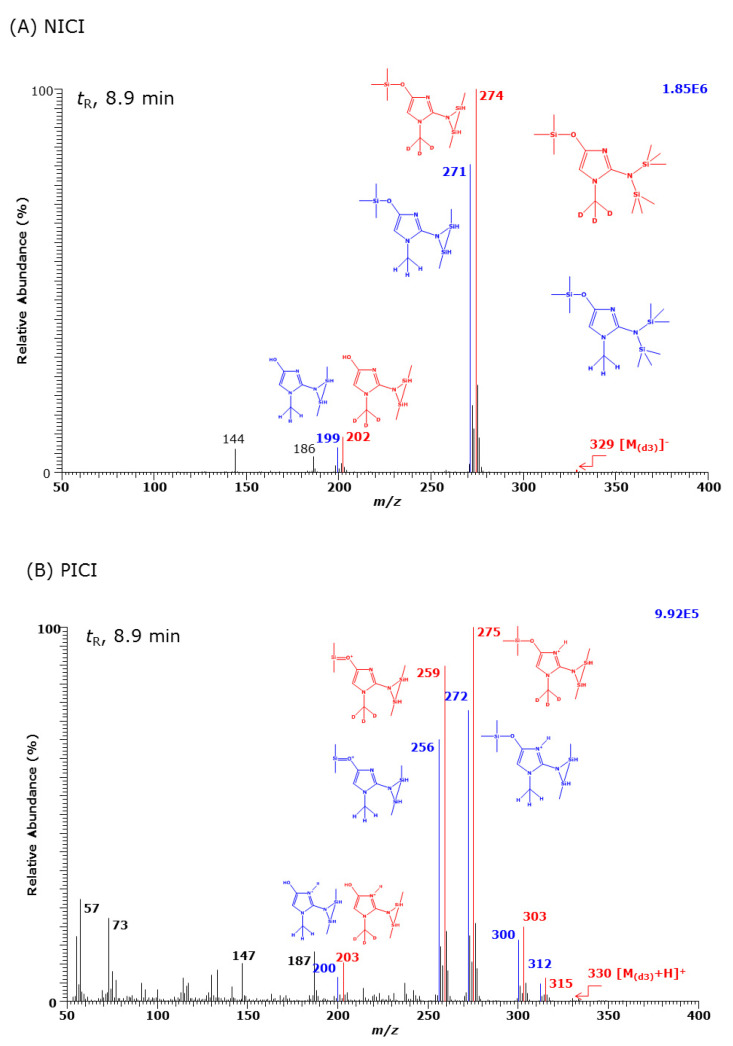
(**A**) Negative-ion chemical ionization (NICI) and (**B**) positive-ion chemical ionization (PICI) GC-MS spectra generated from a mixture of d_0_-creatinine (blue) and d_3_-creatinine (red) after derivatization with *N*,*O*-*tris*(trimethylsilyl)trifluoroacetamide (BSTFA) at 60 °C for 60 min (each 1 nmol injected). The retention time (*t*_R_) of the GC-MS peak was 8.9 min. Insets indicate the proposed structures of the derivatives and ions. See Scheme 2.

Each 100 nmol of d_0_-creatinine and d_3_-creatinine taken from their aqueous solutions were combined, the solvent was evaporated to dryness and derivatized with 100 µL BSTFA as described above. Derivatization resulted in a yellow-colored clear solution. The sample was analyzed by GC-MS in the PICI and NICI mode consecutively by injecting 1-µL aliquots of the BSTFA solutions corresponding each to 1 nmol of d_0_-creatinine and d_3_-creatinine (assuming quantitative derivatization). GC-MS spectra were generated by scanning the quadrupole in the mass-to-charge (*m*/*z*) ratio range of 50–650 and 50–1000 (1 scan per s). We observed two chromatographic peaks with the retention time of 8.6 min and 8.9 min (major peak) and their GC-MS spectra contained paired *m*/*z* values differing by 3 Da due to the three deuterium atoms in methyl group of d_3_-creatinine (Figure 1).

The most intense anions in the NICI mass spectrum (Figure 1A) of the GC peak eluting at 8.9 min were *m*/*z* 271 and *m*/*z* 274 (base peaks). Less intense anions were found at *m*/*z* 199 and *m*/*z* 202, and very weak ions (intensity < 1%) were *m*/*z* 326 and *m*/*z* 329, presumably due to molecular anions of the derivatives (i.e., [M]^−^). These data indicate the presence of the unlabeled methyl group in d_0_-creatinine and of the deuterium-labeled methyl group of d_3_-creatinine in this peak (Figure 1A). The NICI spectrum of this GC peak also contained weak anions at *m*/*z* 144 and *m*/*z* 186 that do not carry the original methyl group of creatinine (Figure 1A). The PICI mass spectrum of the GC peak eluting at 8.9 min contained intense cations at *m*/*z* 272, *m*/*z* 275, *m*/*z* 256 and *m*/*z* 259, less intense cations at *m*/*z* 300 and *m*/*z* 303, weaks ions at *m*/*z* 312 and *m*/*z* 315, and very weak ions (intensity < 1%) at *m*/*z* 330 and *m*/*z* 333, presumably due to the protonated molecules of the derivatives (i.e., [M+H]^+^) (Figure 1B). 

**Scheme 2:**
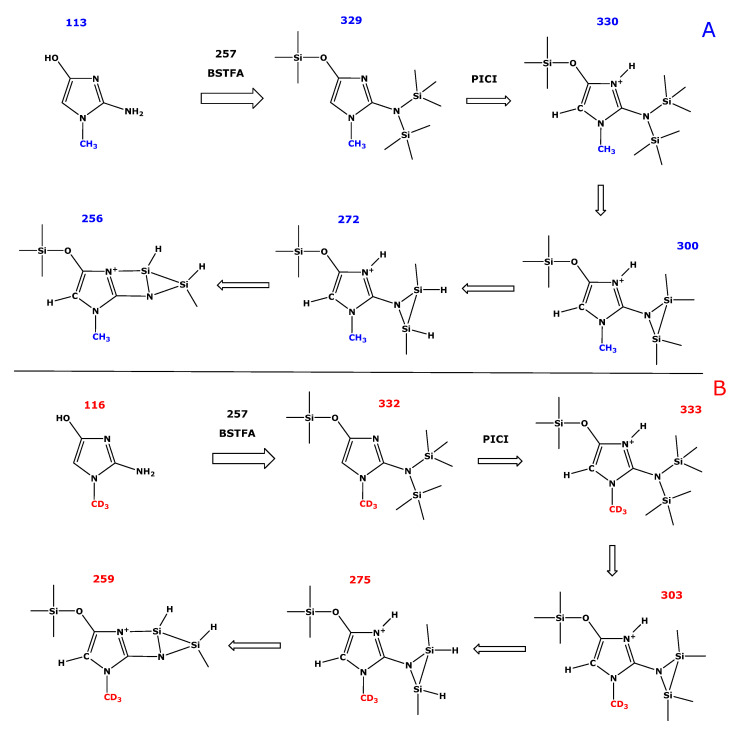
Proposed fragmentation mechanisms for the *N*,*N*,*O*-trimethylsilyl derivatives of d_0_-creatinine (**A**, blue) and d_3_-creatinine (**B**, red) of the GC-MS peak with the retention time of 8.9 min in the PICI mode. The numbers give the molecular weight of the neutral substances and the cations. See Figure 1B.

These data indicate the presence of the unlabeled methyl group in d_0_-creatinine and the deuterium-labeled methyl group of d_3_-creatinine in this peak (Figure 1). Comparison of the total ion intensity in the NICI and PICI mass spectra (1.85 × 10^6^ versus 9.92 × 10^5^, Figure 1) suggests that NICI may allow for a more sensitive detection of creatinine than PICI. Proposed fragmentation mechanisms of the *N*,*N*,*O*-trimethylsilyl derivatives in the PICI are shown in Scheme 2.

The smaller GC peak eluting at 8.6 min had closely comparable NICI and PICI mass spectra to those of the *N*,*N*,*O*-*tris*(trimethylsilyl) derivative (data not shown). These observations suggest that the GC peak with the retention time of 8.6 min is an isomer of the *N*,*N*,*O*-*tris*(trimethylsilyl) derivative of creatinine.

**Figure 2:**
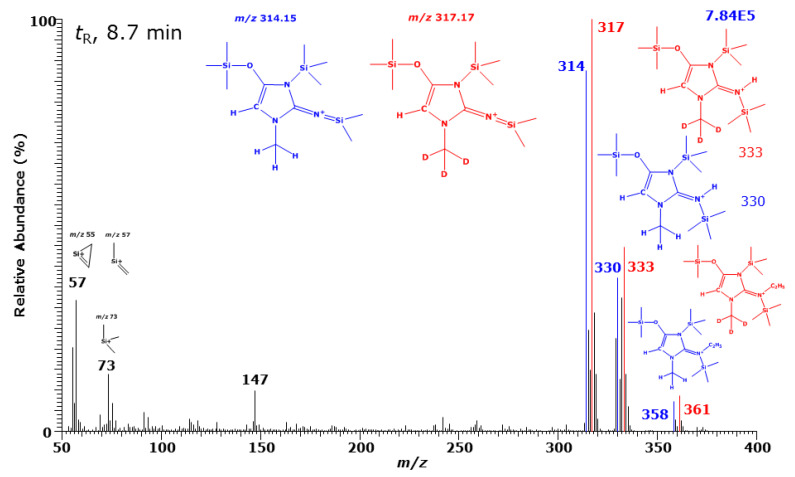
PICI GC-MS spectrum generated from a mixture of d_0_-creatinine (blue) and d_3_-creatinine (red) after derivatization with BSTFA at 60 °C for 60 min. The retention time (*t*_R_) of the GC-MS peak was 8.7 min. Insets indicate the proposed structures for the mass fragments. See also Figure 1B.

The GC peak with the retention time of 8.7 min was only detectable in the PICI mode. The PICI mass spectrum of this peak contained three pairs of cations differing by 3 Da due to the presence of d_3_-creatinine, i.e., *m*/*z* 314 and *m*/*z* 317 (base peaks), *m*/*z* 330 and *m*/*z* 333 ([M+H]^+^), and *m*/*z* 358 and *m*/*z* 361 ([M+C_2_H_4_+H]^+^) (Figure 2). Adducts such as C_2_H_4_ (28 Da) are common in PICI of amines such as dimethyl amine and derive from the reactant gas methane [26]. Presumably, the adduct is on the non-ring amine group. These observations suggest the GC peak eluting at 8.7 min is a creatinine derivative with three trimethylsilyl (TMS) groups, most likely the *N^2^*,*N^3^*,*O^4^*-*tris*(trimethylsilyl) derivative. It cannot ionize in the NICI mode, presumably because of the inability to form anions by loss of an H atom or by capturing an electron due to the lack of electron-capturing atoms and functional groups in the derivative. The cations with *m*/*z* 314 and *m*/*z* 317 seem to be very stable and do not fragment. The cations *m*/*z* 55, *m*/*z* 57, *m*/*z* 73 and *m*/*z* 147 are shared by d_0_-creatinine and d_3_-creatinine and are likely to be associated with the TMS groups (see also [23]) of the derivatives (see also Figure 1B).

### 3.2. Generation of GC-MS Spectra and Characterization of Derivatization Products of d_0_-Creatine

**Figure 3:**
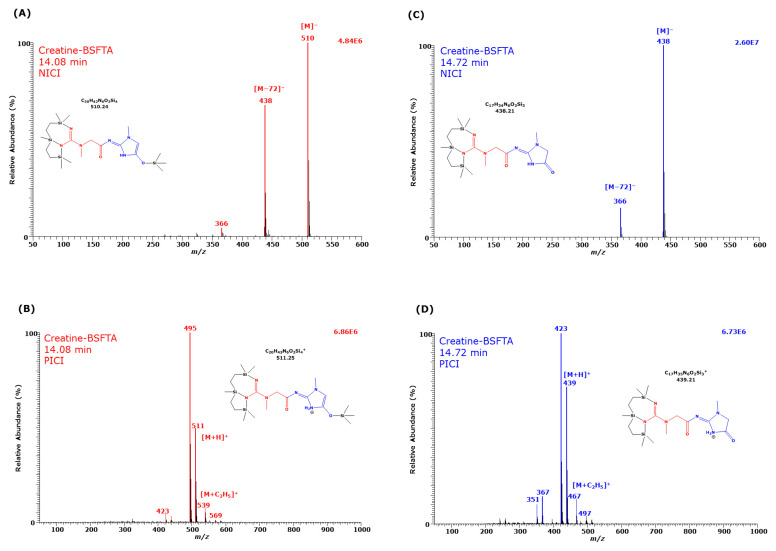
NICI (**A**,**C**) and PICI (**B**,**D**) GC-MS spectra generated from the BSTFA derivatization (60 °C, 60 min) of a freshly prepared solution of d_0_-creatine in deionized water upon its evaporation to complete dryness. The retention times (*t*_R_) of the GC-MS peak were 14.08 min (minor peak, red) and 14.72 min (major peak, blue). Insets indicate the proposed structures for the mass fragments. The same oven column temperature program was used as in Figure 1 and Figure 2.

Derivatization of d_0_-creatine with BSTFA (60 °C, 60 min) resulted in the formation of three GC-MS peaks with the retention times of the d_0_-creatinine. The NICI and PICI mass spectra of these derivatives were virtually identical with those of the d_0_-creatinine derivatives (data not shown). In order to investigate the potential formation of additional derivatives of d_0_-creatine we extended the upper *m*/*z* scanning range to 1000 and the acquisition time to 16 min. We observed two GC-MS eluting at 14.08 min (minor peaks) and 14.72 min (major peaks) in the NICI and PICI mode. The corresponding GC-MS spectra of these d_0_-creatine derivatives and the relatively high difference in their long retention times suggest that these peaks are not derivatives of d_0_-creatine or d_0_-creatinine (Figure 3). A possible explanation could be the formation of a creatinyl-derivative by the reaction of two creatine molecules and/or by the reaction of a creatine molecule and with a molecule of creatinine formed from creatine during the derivatization. The peak with shorter retention time could be due to its TMS ether functionality compared to the keto group.

### 3.3. Standardization of [methylo-^2^H_3_]Creatinine

The isotopic purity of stable isotope-labelled analogs is of particular importance in quantitative analyses [27]. The isotopic purity of the commercially available [*methylo*-^2^H_3_]creatinine was verified as follows.

Nine separate samples containing each 100 nmol of d_0_-creatinine and d_3_-creatinine were derivatized with BSTFA (100 µL) and 1-µL aliquots of their solutions were analyzed by SIM of *m*/*z* 256, *m*/*z* 259, *m*/*z* 271, and *m*/*z* 274 (peak with retention time 8.9 min). Analysis of the sample containing d_0_-creatinine generated a mean PAR of 0.02223 ± 0.00329 (RSD, 15%; *n* = 9) for *m*/*z* 259 to *m*/*z* 256, and a PAR of 0.01302 ± 0.00209 (RSD, 16%; *n* = 9) for *m*/*z* 274 to *m*/*z* 271. Analysis of the samples that contained d_3_-creatinine produced a mean PAR of 0.000588 ± 0.0001411 (RSD, 24%; *n* = 9) for *m*/*z* 256 to *m*/*z* 259 and a mean PAR of 0.01108 ± 0.0002861 (RSD, 2.6%; *n* = 9) for *m*/*z* 271 to *m*/*z* 274. These observations indicate the presence of only very low amounts of d_0_-creatinine in the commercial [*methylo*-^2^H_3_]creatinine and confirm its declared isotopic purity (>99 atom% ^2^H).

### 3.4. Method Linearity, Precision and Accuracy

**Figure 4:**
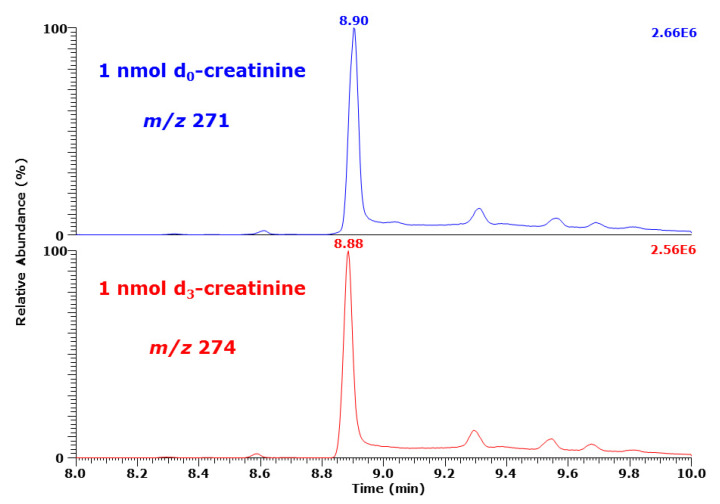
Partial GC-MS chromatograms from the analysis of an equimolar mixture of d_0_-creatinine (blue) and d_3_-creatinine (red) after derivatization with BSTFA at 60 °C for 60 min (each 1 nmol injected). SIM of *m*/*z* 271 for d_0_-creatinine and *m*/*z* 274 for d_3_-creatinine was performed in the NICI mode.

For quantitative analyses of creatinine, we selected the *N*,*N*,*O*-*tris*(trimethylsilyl) derivative of creatinine with the retention of 8.9 min. The structure of this creatinine derivative is most likely *N^2^*,*N^2^*,*O^4^*-*tris*(trimethylsilyl). The structure with the ring-*N^3^* atom of creatinine, which is not derivatized, allows both PICI and NICI. In the NICI mode, SIM of *m*/*z* 271 for d_0_-creatinine and *m*/*z* 274 for d_3_-creatinine was performed. A representative GC-MS chromatogram is shown in Figure 4 and indicates peaks with closely comparable intensity (2.66 × 10^6^ versus 2.56 × 10^6^) due to injection of nominally 1 nmol of each analyte. In the PICI mode, SIM of *m*/*z* 256 and *m*/*z* 272 for d_0_-creatinine and of *m*/*z* 259 and *m*/*z* 275 for d_3_-creatinine was performed. The dwell-time was 108 ms for all ions and the electron multiplier voltage was set to 2025 V.

**Figure 5:**
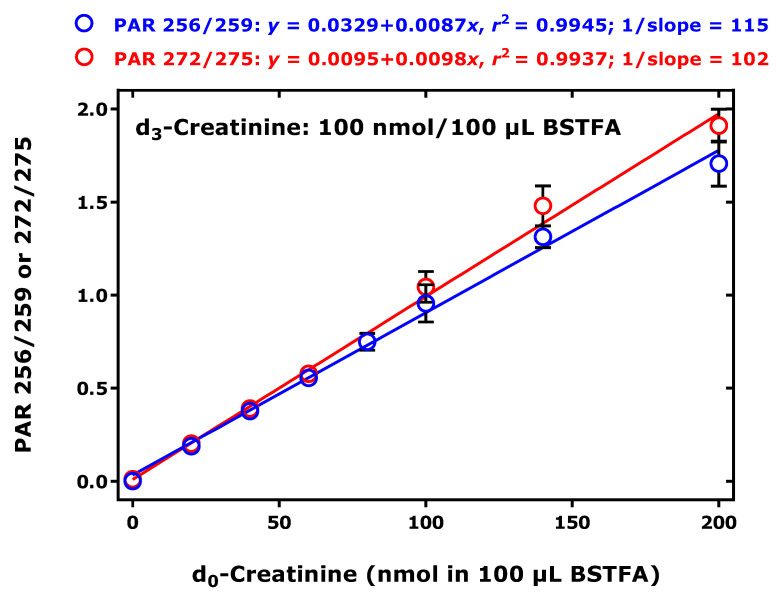
Linear relationships between the peak area ratio (PAR) values and d_0_-creatinine amounts obtained by SIM of *m*/*z* 256 and *m*/*z* 272 for d_0_-creatinine and of *m*/*z* 259 and *m*/*z* 275 for d_3_-creatinine in the PICI mode. The indicated d_0_-creatinine amounts and each 100 nmol d_3_-creatinine were derivatized with BSTFA (100 µL) at 60 °C for 60 min and 1 µL aliquots of the reaction mixture were injected in the splitless mode. Data are shown as mean ± standard deviation (*n* = 9). These analyses were performed by three persons in triplicate for each d_0_-creatinine amount. For more details, see the text.

Stock solutions (each 20 mM) of d_0_-creatinine and d_3_-creatinine were freshly prepared in Ampuwa deionized water. Dilutions of the stock solution of d_0_-creatinine were prepared using Ampuwa water providing d_0_-creatinine concentrations of 0, 2, 4, 6, 8, 10, 14 and 20 mM. Each 10-µL aliquots of these solutions were combined with each 5-µL aliquots of the 20 mM d_3_-creatinine stock solution. After evaporation to dryness under a stream of nitrogen gas, reconstitution of the residue in absolute ethanol and renewed evaporation to dryness, derivatization with 100 µL BSTFA each was performed (60 min, 60 °C). Then. 1-µL aliquots of the samples were injected in the splitless mode and analyzed in the PICI mode by SIM of *m*/*z* 256, *m*/*z* 259, *m*/*z* 272 and *m*/*z* 275. The amounts injected were 1 nmol for d_3_-creatinine in each sample and varying amounts of d_0_-creatinine (i.e., 0.0, 0.2, 0.4, 0.6, 0.8, 1.0, 1.4, 2 nmol). These analyses were performed by three persons in triplicate for each concentration. The precision (relative standard deviation, RSD) ranged between 0.1% and 8.4%. Linear regression analysis between the PAR *m*/*z* 256 to *m*/*z* 259 (*y*) or the PAR *m*/*z* 272 to *m*/*z* 275 (*y*) and the amount of d_0_-creatinine (nmol) (*x*) for all data resulted in straight lines with the regression equations *y* = 0.033 + 0.0087*x* (*r*^2^ = 0.9945) and *y* = 0.001 + 0.0098*x* (*r*^2^ = 0.9937), respectively (Figure 5). The reciprocal values of slopes of the straight lines were 115 nmol and 102 nmol and correspond to the nominal amount of d_3_-creatinine of 100 nmol used in the linearity experiment. Thus, SIM of *m*/*z* 272 and *m*/*z* 275 yields a higher mean accuracy than SIM of *m*/*z* 256 and *m*/*z* 259 (87% vs. 98%) (Figure 5).

### 3.5. Measurement of Creatinine in Human Urine in the NICI Mode

**Figure 6:**
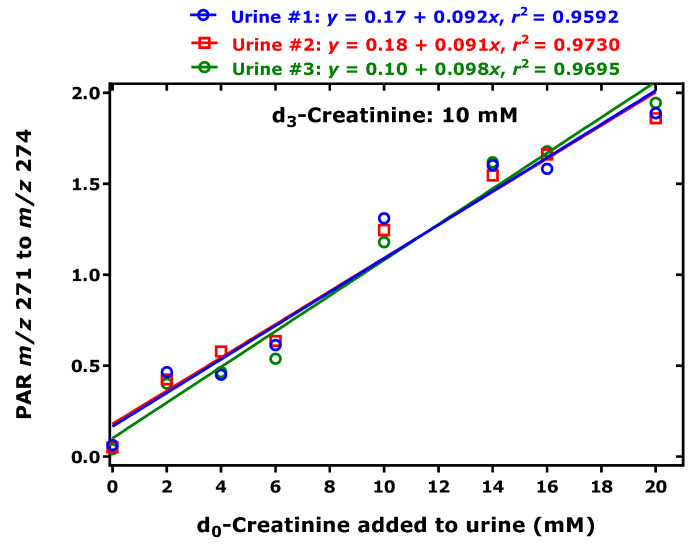
Linear relationships between the peak area ratio (PAR) values and the varying d_0_-creatinine concentrations added to human urine samples donated by three healthy volunteers and regression equations. d_3_-Creatinine was added at the fixed concentration of 10 mM and served as the internal standard. SIM of *m*/*z* 271 and *m*/*z* 274 for d_0_-creatinine and d_3_-creatinine was performed in the NICI mode, respectively. The analyses were performed by the three persons who performed the analyses shown in Figure 5. For more details, see the text.

The method was validated in human urine samples in the NICI mode by the three persons who performed the experiment described above. Three healthy volunteers (#1, #2, #3) donated urine samples by spontaneous micturition. To 10-µL urine aliquots, d_0_-creatinine was added to reach final added concentrations of 2, 4, 6, 10, 14 and 20 mM. d_3_-Creatinine was also added to these samples to reach a fixed concentration of 10 mM in each urine sample. After evaporation to dryness under a stream on nitrogen gas, reconstitution of the residues in 100 µL aliquots of absolute ethanol and renewed evaporation to dryness, derivatization each with 100 µL BSTFA was performed (60 min, 60 °C) and 1-µL aliquots were injected and analyzed by SIM of *m*/*z* 271 and *m*/*z* 274 in the NICI mode. Linear regression analysis between the PAR of *m*/*z* 271 to *m*/*z* 274 (*y*) and the concentration of d_0_-creatinine (mM) (*x*) resulted in straight lines (Figure 6). The reciprocal slope values of the straight lines were 10.9 mM for urine #1, 11.0 mM for urine #2, and 10.2 mM for urine #3. Based on the nominal concentration of d_3_-creatinine of 10 mM in the urine samples, the mean accuracy is calculated to be 109%, 110% and 102% in the three human urine samples in the concentration range investigated. The *y* axis intercept values indicate mean basal creatinine+creatine concentrations of 1.7, 1.8 and 1 mM, respectively.

### 3.6. HPLC Analysis of Creatinine in HCl Solutions of Creatine

**Figure 7:**
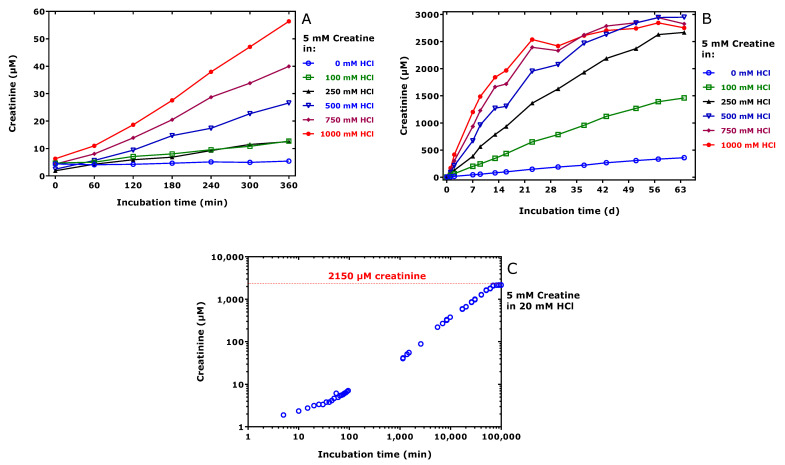
(**A**–**C**) Creatinine formed upon incubation of 5 mM creatinine in deionized water and in the indicated HCl solutions for the indicated times at room temperature (22–25 °C). Analyses were performed by HPLC with UV absorbance detection at 210 nm. Note the double decadic logarithmic scale in panel (**C**).

The aim of these analyses was to estimate the extent of formation of creatinine from creatine and creatine-phosphate in hydrohloric acid solutions of varying molarity and incubation time at room. Linear relationships between the response (*y*), i.e., peak area, mAU×min at 210 nm, and the creatinine concentration in µM (*x*) was observed: *y* = 7.4 + 4.92 *x*, (*r*² = 0.9999) (range, 0–1000 µM). This regression equation was used to measure the concentration of creatinine in creatine solutions in hydrochloric acid (Figure 7). The concentration of creatinine in freshly prepared 5000 µM creatine solutions ranged between 2 and 7 µM and increased with increasing HCl molarity and incubation time up to 56 µM at 1 M HCl and 360 min (Figure 7A) and up to 2500–3000 µM after 63 days in 250–1000 mM HCl solutions (Figure 7B). The sigmoidal creatinine-incubation time profile in a 5000 µM solution of creatine in 25 mM HCl is shown in (Figure 7C). The highest creatinine concentration was determined to be 2150 µM after 69 days. Creatine-phosphate was found to be stable in deionized water. Similar experiments with HCl-solutions of creatine-phosphate did not result in formation of considerable amounts of creatinine (data not shown). Except for creatine and creatinine we did not detect appearance of additional peaks within HPLC run time of 5 min and UV absorbance detection at 210, 232 and 250 nm.

## 4. Discussion

Silylation is one of the most widely used derivatization reaction in analytical chemistry, notably in GC-based methods. Silylation reagents such as BSTFA and MSTFA are not specific, but react with different functionalities of organic compounds, especially of hydroxyl and amine groups, to form *O*- and *N*-trimethylsilyl derivatives [23]. Such derivatives are volatile and thermally stable in non-aqueous systems, best properties in GC-based analytical methods.

Creatinine, 2-amino-1-methyl-5*H*-imidazol-4-one (Scheme 1), is an endogenous substance, the final metabolite of creatine catabolism. Creatinine can be formed chemically from creatine by acid-catalyzed cyclization (Scheme 1). The most significant field of interest in creatinine is Clinical Chemistry. Serum creatinine serves as an indicator of kidney function. Urinary creatinine is of particular importance in clinical, pharmacological and epidemiological studies, where biomarkers must be measured in urine collected from spontaneous micturition, i.e., when the urine volume and the time between two urine collections are unknown. This particular importance is because creatinine is excreted in the urine with a relatively constant rate primarily via glomerular filtration mainly depending on age and gender. The great interest in creatinine in various disciplines led to the development of many analytical methodologies based on different principles. As an organic amine, derivatization of creatinine improves its physicochemical properties so that its analysis becomes feasible by GC also coupled with mass spectrometry (MS) [3,4,5,6,7,8,9,10,11,12,13,14,15,16,17,18,19,20,21,22,23]. Thus, GC-MS was used several decades ago for the quantitative measurement of creatinine in biological samples including serum and urine using stable isotope-labelled analogs of creatinine [20,21,22].

Using trimethylsilylation (no conditions reported), Lawson found by GC-MS and EI that creatinine is converted to a single derivative, which was identified as the *N*,*N*,*O*-TMS derivative [20]. The EI mass spectrum of this derivative contained two ions at *m*/*z* 329, which is the the molecular radical cation [M]^●+^, and *m*/*z* 314 due to the loss of methyl radical ([M‒CH_3_]^+^) from one of the three TMS groups [20]. This derivative obviously corresponds to the derivatives of d_o_-creatinine of d_3_-creatinine in our study with the retention time of 8.9 min. Siekmann extracted creatinine from human serum samples by cation-exchange resin Ag 50W-X2, derivatized by *N*-methyl-*N*-trimethylsilyl-trifluoroacetamide (MSTFA) in anhydrous pyridine (1:1, *v*/*v*) by heating (40 min, 60 °C) [21]. Siekmann reported on the formation of a single GC-MS peak (by SIM), of which the EI spectrum was very similar to that reported by Lawson [20], supporting the formation of a *N*,*N*,*O*-TMS derivative of creatinine. Neither Lawson nor Siekmann reported in their papers analogous analyses with creatine.

**Scheme 3:**
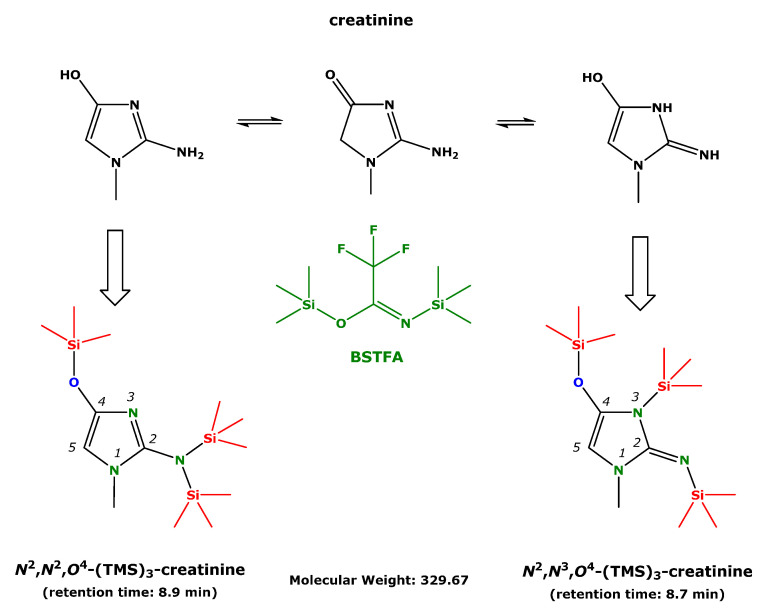
Proposed chemical structures for two derivatives of creatinine formed by its reaction with *N*,*O*-*tris*(trimethylsilyl)trifluoroacetamide (BSTFA) at 60 °C for 60 min in pure BSTFA.

Our observations strongly suggest that derivatization of creatinine (60 min, 60 °C) with pure BSTFA, i.e., in the absence of any solvents such as pyridine generates at least three derivatives. The derivative eluting at 8.9 min is most likely *N*^2^,*N*^2^,*O*^4^-*tris*(trimethylsilyl)-creatinine, identical with that proposed by Lawson [20] and Siekmann [21]. The second major derivative formed under the same derivatization conditions is most likely *N^2^*,*N^3^*,*O^4^*-*tris*(trimethylsilyl)-creatinine with the retention time of 8.7 min (Scheme 3). This derivative has not been reported thus far. *N^2^*,*N^3^*,*O^4^*-*tris*(Trimethylsilyl)-creatinine elutes in front of *N^2^*,*N^2^*,*O^4^*-*tris*(trimethylsilyl)-creatinine presumably because all derivatizable N atoms of creatinine are derivatized.

Our study also strongly suggests that derivatization of creatine with pure BSTFA under same conditions (60 min, 60 °C) generates the same two derivatives eluting at 8.7 min and 8.9 min. The results from HPLC analyses of creatine solutions in deionized water and hydrochloric acid solutions suggest that creatine cyclizes to form creatinine, yet a very low extent. One may therefore assume that the derivatives *N^2^*,*N^3^*,*O^4^*-*tris*(trimethylsilyl) and *N^2^*,*N^2^*,*O^4^*-*tris*(trimethylsilyl) are formed during the BSTFA derivatization step.

**Scheme 4:**
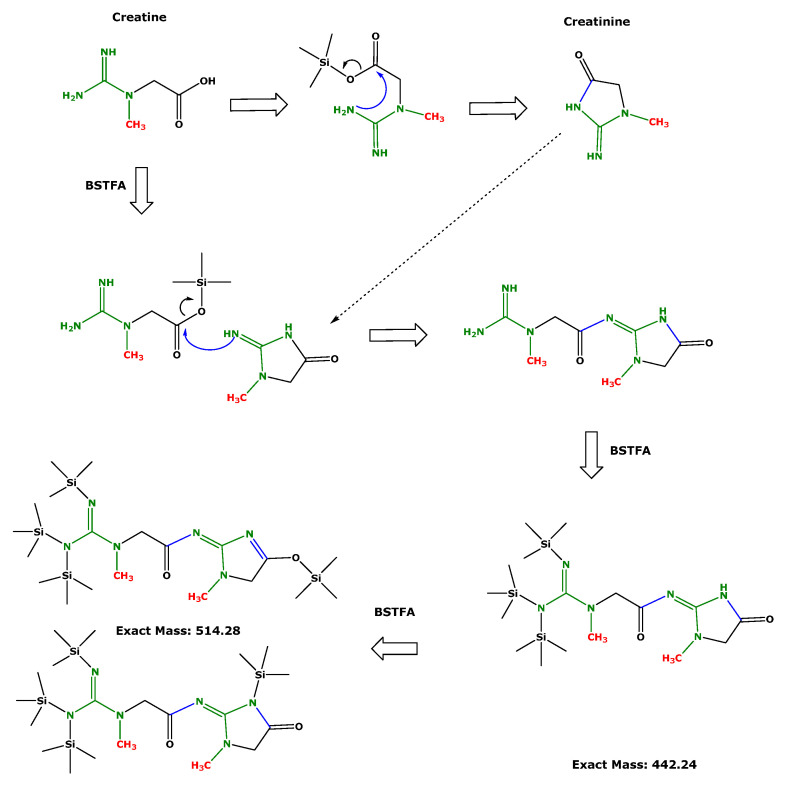
Proposed chemical structures for the formation of creatinyl-creatinine derivatives from the derivatization of creatine with pure *N*,*O*-*tris*(trimethylsilyl)trifluoroacetamide (BSTFA) at 60 °C for 60 min. See the NICI and PICI mass spectra of these derivatives in Figure 3.

To the best of our knowledge, the present study is the first to demonstrate the formation of two new derivatives from creatine via BSTFA derivatization (60 min, 60 °C). These derivatives emerge from the column 5 to 6 min later than the above-mentioned derivatives of creatinine and creatine. Our study strongly suggests that both late-eluting TMS derivatives stem from a creatine-creatinine adduct. As no creatinine was initially present in the creatine sample, the detected creatine-creatinine is likely to have been formed by alternative mechanisms. One possible mechanism could involve formation of the *O*-TMS ester of creatine (Scheme 4), which is likely to be formed more rapidly and to a higher extent than the *N*-TMS [24]. Subsequently, free amine groups may attack the chemically activated carboxylic group to make the creatinine residues. Thus far, only one group has reported on the synthesis of creatinyl-amino acids derivatives such as creatinyl-glycine, which has been reported to be neuroprotective [28]. In the NICI and PICI mass spectra of these derivatives we obtained mass fragments being each by 4 Da (see Figure 3). An explanation for this finding could be loss of 4 H atoms in total on the three trimethylsilyl groups of the terminal guanidine group. We do not know whether this results from the derivatization or ionization irrespective of the ionization mode. Such a phenomenon has not been reported thus far. Yet, there is an indication that this may occur in *N*,*N*-di-trimethylsilyl derivatives [29,30,31]. Thus, in the EI mass spectra of the per-trimethylsilylated 1-phosphono-2-amino-ethane (MW 413) and *O*-phosphorylethanolamine (MW 429) the cation *m*/*z* 174 was observed, which was assigned to [CH_2_=N(Si(CH_3_)_3_)_2_]^+^. These spectra also contained *m*/*z* 172 with intensity ratio of 2:1. A possible structure for *m*/*z* 172 could be [CH_2_=N(Si(CH_3_)_2_CH_2_)_2_]^+^. 

Silylation of this compound with a perdeuterated silylation reagent shifted these cations to *m*/*z* 192 and *m*/*z* 188 [30], strongly supporting the bridging of two methyl groups of the neighboring TMS groups on the amine group. BSTFA and other silylation agents can react with various functionalities [23], including acetamide groups such as that of acetaminophen (paracetamol) to generate its *O*,*O*-di-TMS derivative [31], and their derivatives undergo multiple fragmentations and rearrangements during ionization such as EI [32].

Pentafluoropropionic anhydride (PFPA) is another useful derivatization reagent in GC-MS. Like BSTFA, PFPA also reacts with amine, hydroxylic and carboxylic groups for instance of amino acids [33]. The *N*-pentafluoropropionyl derivatives are considerably more stable than the *O*-pentafluoropropionyl derivatives [34]. As BSTFA derivatization does not allow discrimination between creatinine and creatine, we tested the utility of PFPA. Under conditions previously reported for amino acids [25,32], i.e., heating the analytes in PFPA-ethyl acetate (1:4, *v*/*v*; 65 °C, 30 min), we observed each only one peak from creatine, d_0_-creatinine and d_3_-creatinine. The GC-NICI-MS spectra of creatine and d_0_-creatinine derivatives were virtually identical: *m*/*z* 221 (6 %; [M−HF−H_2_O]^−^), 239 (100 %; [M−HF]^−^) and *m*/*z* 259 (6 %; [M]^−^; C_7_H_6_F_5_N_3_O_2_); the GC-MS spectrum of the d_3_-creatinine derivative eluted a few seconds earlier: *m*/*z* 224 (6 %), 242 (100 %) and *m*/*z* 262 (6 %). These results indicate the formation presumably of *N*^2^-pentafluoropropionyl from both, creatine and creatinine. These observations suggest that PFPA reacts with the carboxylic group of creatine to form the mixed anhydride. Subsequently, the *N*^2^-imine group attacks intra-molecularly the carboxylic group, with pentafluoropropionic acid leaving the molecule, analogous to the BSTFA derivative of creatine.

As far we are informed, creatinine has not be measured by GC-MS in human urine after derivatization with BSTFA. Our study indicates that creatinine can be quantified precisely and accurately by GC-MS in only 10-µL aliquots of human urine using d_3_-creatinine as internal standard in relevant concentration ranges. The method does not require any organic solvent or base like pyridine for derivatization and/or extraction for GC-MS analysis. Excess BSTFA serves as a solvent, in which the TMS derivatives are readily soluble, yet no other charged endogenous constituents present in urine. As BSTFA is highly reactive towards numerous substances [23], it is possible that many endogenous substances also form volatile TMS derivatives that do not accumulate in the GC column.

Currently available data suggest that specific measurement of creatinine in urine and other biological samples is possible by using 2,3,4,5,6-pentafluorobenzyl (PFB) bromide (PFB-Br) in aqueous acetone (60 min, 50 °C) [34]. Creatinine reacts with PFB-Br to form a single derivative, i.e., *N*^2^-PFB-creatinine. Interestingly, we found that the *N*^2^-PFB-derivatives of d_0_-creatinine and d_3_-creatinine react with PFPA (65 °C, 30 min) to form their *N*^2^-PFB,*N*^3^-PFP derivatives (MW=439.21, C_14_H_7_F_10_N_3_O_2_; MW=442.23, C_14_H_4_D_3_F_10_N_3_O_2_, respectively) with relative retention time of 1.29 with respect to the *N*^2^-PFB-derivatives.

## 5. Conclusions

BSTFA is known for many decades as a useful derivatization reagent for the GC-MS analysis of creatinine, but its utility to measure creatinine in human urine has not been reported thus far. This study investigated the derivatization of creatinine and its precursor creatine with BSTFA. Both substances react with BSTFA (60 °C, 60 min) to form three derivatives of virtually identical structures. Creatinine and creatine were found to react with PFPA (65 °C, 30 min) to form a single *N*-pentafluoropropionyl derivative. These observations indicate that BSTFA and PFPA is much more effective in the conversion of creatine to creatinine than lowering the pH by inorganic acids such as hydrochloric acid. Our findings suggest that BSTFA and PFPA are not useful for the simultaneous measurement of creatinine and creatine. The *N*,*N*,*O*-trimethylsilyl derivative of creatinine and creatine with the retention time of 8.9 min is useful for their quantitative measurement in human urine both in the NICI and PICI mode using trideuteromethyl creatinine as internal standard. Under the same derivatization conditions, creatine reacts with BSTFA and forms two creatinyl-creatine derivatives with retention times of 14.07 min and 14.72 min, suggesting intermediate formation of creatinine and its conjugation with creatine. Our results show that methyl groups of the TMS residues react to form -CH_2_-CH_2_-bridges and are supported by previous reports on alkyl amines. Such a cyclization reaction is more likely to occur during EI, PICI and NICI, rather than during the derivatization with BSTFA. The possibility that creatinine, but not creatine, reacts with PFB-Br and the *N*^2^-PFB-creatinine derivative reacts with PFPA to form *N*^2^-PFB,*N*^3^-PFP-creatinine offers the possibility to measure biological creatinine and creatine simultaneously by GC-MS. This could be of particular importance in the area of Clinical Chemistry and in clinical trials.

## Data Availability

The study did not report any data.

## Sample Availability

Not available.
